# Supplementary material for: Defeat, Entrapment, and Hopelessness: Clarifying Interrelationships between Suicidogenic Constructs
Source: Int J Environ Res Public Health. 2022 Aug 24;19(17):10518. doi: 10.3390/ijerph191710518 (PMC9517901; doi:10.3390/ijerph191710518)
Supplement: Supplementary file 1 [file ijerph-19-10518-s001.zip › ijerph-1858649-supplementary.pdf]

Online Supplement Table S1. Independent samples t-test of mean differences between study variables nested within school.									
	**Sample**	**N**	**M**	**SD**	***t***	**df**	**Sig.**	Mean diff.	Std. Err. diff.
Defeat	1	210	17.23	13.05	-9.66	342	.000	-13.76	1.42
	2	134	31.49	12.60					
EE	1	210	8.05	9.20	-8.91	342	.000	-9.07	1.02
	2	134	17.12	9.21					
IE	1	210	5.24	6.55	-9.16	342	.000	-6.94	.76
	2	134	12.18	7.31					
BHS Affect	1	210	6.60	5.13	-7.13	342	.000	-4.32	.61
	2	134	10.93	5.99					
BHS Mot.	1	210	6.10	6.30	-2.48	342	.014	-1.73	.70
	2	134	7.83	6.39					
BHS Cog.	1	210	7.98	4.96	-4.67	342	.000	-2.66	.57
	2	134	10.63	5.44					
BHS Total	1	210	20.68	14.70	-5.23	342	.000	-8.71	1.67
	2	134	29.39	15.71					

Note. EE=external entrapment, IE=internal entrapment, BHS=Beck Hopelessness Scale.

Online Supplement Table S2.

Table S2. Items from the Entrapment Scale, Defeat Scale, and Beck's Hopelessness Scale.

Item	*1*	*2*	*3*	*4*	*h^2*	*Skew*	*Kurt.*
DS1	.774	.376	.516	.407	.62	.311	-.928
DS2[†]	.571	354	.591	.-	.62	.175	-.731
DS3	.841	.402	585	.443	.73	.362	-.841
DS4[†]	.584	.384	.604	-	.71	-.190	-.717
DS5	.820	.434	.494	.417	.70	.513	-.746
DS6	.764	.395	.441	.429	.62	.441	-.795
DS7	.791	.407	.548	.500	.67	.306	-.767
DS8	.824	.403	.533	.472	.70	.318	-.814
DS9[†]	.445	.302	.532	-	.41	.191	-.427
DS10	.843	.501	.524	.491	.76	.649	-.363
DS11	.831	.471	.465	.481	.76	.654	-.505
DS12	.778	.560	.518	.434	.73	.953	-.077
DS13	.798	.418	.543	.434	.68	.684	-.512
DS14	.827	.384	.491	.440	.69	.430	-.777
DS15	.792	.430	.451	.500	.65	.498	-.667
DS16	.738	.479	.517	.412	.68	.926	.075
ES1(external)	.738	.420	.541	.717	.74	.798	-.635
ES2(external)	.767	.385	.546	.624	.73	.334	-1.371
ES3(external)	-	-	-	.547	.36	1.798	2.473
ES4(external)	.725	.361	.481	.659	.69	.405	-1.362
ES5(external)	.757	.471	.558	.682	.70	.702	-.692
ES6(external)	.726	.322	.506	.658	.65	.371	-1.151
ES7(external)	.633	.463	.498	.697	.66	1.238	.444
ES8(external)	.509	.354	-	.711	.53	1.190	.180
ES9(external)	.635	.350	.486	.685	.59	.771	-.771
ES10(external)	.555	.328	.358	.795	.68	1.223	.364
ES11(internal)	.788	.398	.553	.505	.80	.748	-.900
ES12(internal)	.749	.449	.552	.599	.67	.954	-.261
ES13(internal)	.842	.382	.532	.573	.81	.302	-1.415
ES14(internal)	.827	.380	.525	.571	.80	.727	-.868
ES15(internal)	.779	.385	.526	.592	.79	.489	-1.309
ES16(internal)	.770	.509	.547	.634	.71	1.027	-.343
BHS1(feeling)[†]	.678	.463	.794	.398	.71	.562	-.801

BHS2(motivation)	.411	.670	.438	-	.49	1.883	2.890
BHS3(feeling)	.419	.310	.586	-	.38	.480	-.735
BHS4(expect)	.330	.405	-	-	.36	.101	-1.191
BHS5(feeling)†	.499	.369	.617	.447	.48	.369	-.919
BHS6(feeling)†	.531	.499	.811	-	.69	.596	-.750
BHS7(expect)	.505	.762	.544	.313	.65	1.373	1.199
BHS8(expect)†	.463	-	.623	-	.42	-.052	-1.262
BHS9(motivation)	.365	.652	.301	-	.45	.989	.093
BHS10(expect)†	.416	.348	.632	.350	.44	.343	-.751
BHS11(motivation)	.411	.763	.452	.303	.60	1.584	1.715
BHS12(motivation)	.461	.685	.418	.342	.53	.798	-.551
BHS13(feeling)†	-	.343	.604	-	.49	.392	-.959
BHS14(expect)	.530	.750	.396	.444	.78	.828	-.287
BHS15(feeling)†	.616	.541	.854	.355	.65	.448	-1.065
BHS16(motivation)	-	.784	.336	-	.76	1.796	2.413
BHS17(motivation)	.328	.857	.417	-	.86	1.612	1.762
BHS18(expect)	.510	.688	.541	-	.69	.579	-.910
BHS19(feeling)†	.569	.448	.777	.379	.67	.558	-.710
BHS20(motivation)	.300	.804	.342	-	.68	1.792	2.694
Initial Eigenvalues	24.54	3.75	2.32	1.69	-	-	-
Extraction Eigenvalues	24.21	3.40	1.90	1.28	-	-	-
Initial % of Variance	47.19	7.22	4.46	3.24	62.11	-	-
Extracted % of Variance	46.56	6.54	3.66	2.46	59.22	-	-

Note: †= reverse coded item. Loadings above .40 are significant. Factor loadings < .30 are suppressed.
